# pH-sensitive release of nitric oxide gas using peptide-graphene co-assembled hybrid nanosheets

**DOI:** 10.1016/j.niox.2024.04.008

**Published:** 2024-04-15

**Authors:** Tanveer A. Tabish, Jiamin Xu, Christopher K. Campbell, Manzar Abbas, William K. Myers, Pravin Didwal, Dario Carugo, Fang Xie, Mark J. Crabtree, Eleanor Stride, Craig A. Lygate

**Affiliations:** a Division of Cardiovascular Medicine, Radcliffe Department of Medicine, British Heart Foundation (BHF) Centre of Research Excellence, University of Oxford, Oxford, OX3 7BN, United Kingdom; b Department of Materials and London Centre for Nanotechnology, Imperial College London, London, SW7 2AZ, United Kingdom; c Nuffield Department of Orthopaedics, Rheumatology and Musculoskeletal Sciences (NDORMS), The Botnar Research Centre, University of Oxford, Oxford, OX3 7LD, United Kingdom; d Department of Chemistry, Khalifa University of Science and Technology, P.O. Box, 127788, Abu Dhabi, United Arab Emirates; e Centre for Advanced Electron Spin Resonance (CAESR), Inorganic Chemistry Laboratory, Department of Chemistry, University of Oxford, Oxford, OX1 3QR, United Kingdom; f Department of Materials, University of Oxford, Parks Road, Oxford, OX1 3PH, United Kingdom; g Department of Biochemical Sciences, School of Biosciences and Medicine, University of Surrey, Guildford, GU2 7XH, United Kingdom; h Institute of Biomedical Engineering (IBME), Department of Engineering Science, University of Oxford, Oxford, OX3 7LD, United Kingdom

**Keywords:** Dipeptides, Graphene oxide, Nanohybrids, Co-assembly, Nitric oxide release, pH responsiveness, Cardiovascular disease

## Abstract

Nitric oxide (NO) donating drugs such as organic nitrates have been used to treat cardiovascular diseases for more than a century. These donors primarily produce NO systemically. It is however sometimes desirable to control the amount, location, and time of NO delivery. We present the design of a novel pH-sensitive NO release system that is achieved by the synthesis of dipeptide diphenylalanine (FF) and graphene oxide (GO) co-assembled hybrid nanosheets (termed as FF@GO) through weak molecular interactions. These hybrid nanosheets were characterised by using X-ray diffraction, Raman spectroscopy, Fourier transform infrared spectroscopy, zeta potential measurements, X-ray photoelectron spectroscopy, scanning and transmission electron microscopies. The weak molecular interactions, which include electrostatic, hydrogen bonding and π-π stacking, are pH sensitive due to the presence of carboxylic acid and amine functionalities on GO and the dipeptide building blocks. Herein, we demonstrate that this formulation can be loaded with NO gas with the dipeptide acting as an arresting agent to inhibit NO burst release at neutral pH; however, at acidic pH it is capable of releasing NO at the rate of up to 0.6 μM per minute, comparable to the amount of NO produced by healthy endothelium. In conclusion, the innovative conjugation of dipeptide with graphene can store and release NO gas under physiologically relevant concentrations in a pH-responsive manner. pH responsive NO-releasing organic-inorganic nanohybrids may prove useful for the treatment of cardiovascular diseases and other pathologies.

## 1 Introduction

Nitric oxide (NO) is a signaling molecule that plays an important role in many physiological processes, such as vascular relaxation, neurotransmission, immunological control, and inflammatory responses [1, 2]. For example, NO is a crucial vasodilatory substance in blood vessels where it is produced continuously by vascular endothelial cells [3]. Dysregulation of NO is a common feature in hypertension, angina, heart failure and in the response to vascular injury. For this reason, the exogenous administration of systemic NO donors, such as organic nitrates, have been used to treat cardiovascular diseases for more than a century [4]. At other times, the delivery of NO is necessarily more discrete, for example, immune cells generate large bursts of NO locally in order to kill invading pathogens [2]. An emerging application is the prevention of myocardial ischaemia-reperfusion (I/R) injury, where blood flow in the coronary arteries is impaired resulting in heart attack. Standard clinical treatment is to re-open the diseased artery to salvage the affected myocardium, however, part of the damage is caused by reperfusion of oxygen rich blood, which stimulates mitochondria within the cells to produce a large burst of cytotoxic reactive oxygen species (ROS). NO delivered at the point of reperfusion can prevent this by inhibiting cellular respiration, but this effect must be temporary, since respiration is required to generate cellular energy [5–7]. Hence, there are times when it is necessary to deliver just the right amount of NO to the right location at the right time.

In the present study, we hypothesised that nanomaterials conjugated with NO sources could provide a suitable and tuneable NO delivery platform. A number of examples have previously been described, e.g. Schoenfisch and co-workers have developed a wide-range of nanoparticles (NPs) including silica, dendrimers, gold, and other polymeric NPs for the storage and release of NO, which have primarily been tested for the treatment of bacterial infections [8]. However, the conjugation of NO donors on NP surfaces poses challenges in controlling or preventing premature release; therefore, NO delivery systems have been designed that are sensitive to environmental conditions. For example, Lee et al. developed a pH-responsive NO-releasing calcium carbonate (CaCO_3_) NPs for wound healing, demonstrating the release of NO over 60 h [9]. To the best of our knowledge, short-term pH responsive NO release has not been reported yet, but this would be an attractive property for targeting myocardial I/R injury, since the intracellular pH of ischaemic cardiomyocytes becomes acidic [10].

Graphene has received a lot of attention over recent years due to its well-defined sheet-like structure and exceptionally high specific surface area, which can provide a large storage capacity for drugs, biomolecules or gases [11]. In particular, graphene oxide (GO) holds promise for a wide range of healthcare applications, offering unique opportunities to advance diagnostics, therapeutics, and regenerative medicine. For example, GO can serve as an effective drug delivery platform due to its ability to adsorb and carry therapeutic payloads, including small drugs, proteins, and nucleic acids [12,13]. Functionalisation of GO with targeting ligands or stimuli-responsive moieties allows for targeted and controlled drug release, enhancing therapeutic efficacy while minimising side effects. One potential approach is to combine the storage capacity of graphene with peptides, such as diphenylalanine (FF), that would allow controlled release of NO gas. FF has good biocompatibility having been derived from an endogenous polypeptide [14] and is a self-assembling peptide, a novel class of nanostructured biomaterials capable of organising into well-ordered and uniform hierarchical architectures [15,16]. These non-covalent interactions play a crucial role in driving the self-assembly process, resulting in the formation of various nanostructures such as NPs, nanotubes, and nanosheets, depending on the specific synthesis conditions [17,18]. Under certain conditions, FF spontaneously forms self-assembled hybrids through molecular interactions such as hydrogen bonding, electrostatic, and π-π interactions. By changing the assembly conditions (pH, solvent types, peptide concentration), or introducing nanomaterials (with varying size, shape, and functional groups), a reversible shape transition between self-assembled dipeptide and vesicle-like structures can be achieved [19,20].

Herein, we describe the synthesis and characterisation of co-assembled FF/graphene oxide (FF@GO) hybrid nanosheets for pH sensitive release of NO gas. We perform chemical and morphological characterisation under acidic, neutral, and basic conditions, and demonstrate that sustained real-time release of NO occurs predominantly at acidic pH. This study represents proof-of-concept for a novel type of pH-sensitive organic-inorganic hybrid nanosystem for controlled delivery of NO, which may have a range of biomedical applications.

## 2 Results and discussion

**Fig. 1:**
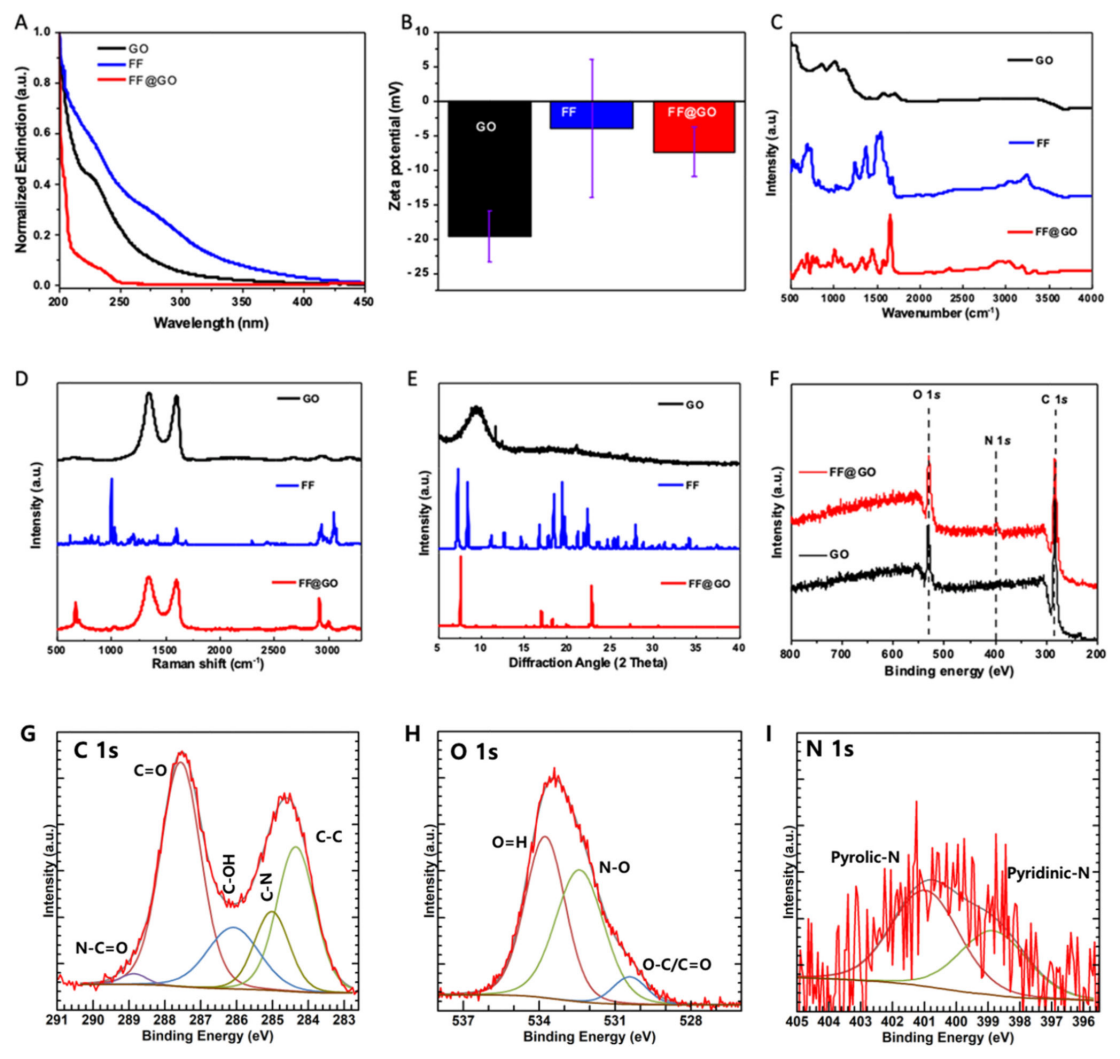
Basic characterisation of graphene oxide (GO), FF peptide and self-assembled FF@GO hybrid nanosheets. (A) UV/vis absorbance spectra recorded in deionised (DI) water, (B) zeta potential measurements (C) FTIR (D) Raman spectra, (E) XRD, (F) XPS survey spectra of GO and FF@GO. (G–I), High-resolution XPS spectra of FF@GO, showing typical C 1s region (G), O 1s region (H), and N 1s region (I).

GO was prepared from graphite flakes by Hummer’s modified method as previously reported by us [19,21–23]. GO is a highly oxidised form of graphene, characterised by hydroxyl groups on basal plane and carboxylic acid functional groups on the edges. Both these functional groups can be deprotonated under basic conditions and thus we utilised the deprotonated form of GO for the co-assembly with dipeptide FF to make hybrid nanosheets. To understand the mechanism behind the formation of hybrid nanosheets, we used ultraviolet–visible (UV–Vis) spectrophotometry and collected the absorption spectra of GO, FF, and FF@GO (Fig. 1 A). There was a blue shift in the absorption of FF@GO hybrid nanosheets compared to FF or GO on their own, which indicates H-aggregation type of assembly due to face-to-face stacking of components [24], thus, π–π interactions contribute to the formation of hybrid nanosheets. Electrostatic interaction could also play a role in the co-assembly of nanosheets because the surface potential of FF@GO was decreased to −8.40 mV compared to that of pure GO at −18.73 mV (Fig. 1B). It is evident that carboxylic acid and hydroxyl groups of GO can be deprotonated to bring the negative charge and the *N*-terminus of FF can be protonated, but FF has surface potential close to zero due to its zwitterionic form. However, it is most likely that the protonated terminus of FF (positively charged) can interact with the negatively charged GO through electrostatic interaction, which may explain the lower surface potential of nanosheets. Thus, a synergistic effect of π–π stacking between aromatic moieties and electrostatic interaction is central to making co-assembled hybrid nanosheets.

To further elucidate the chemistry behind the formation of nanosheets, we carried out Fourier Transform Infra-Red (FTIR) spectroscopy from 500 to 4000 cm^−1^. The FTIR region 3500–3200 cm^−1^ is important for assessing N–H stretching vibrations, which provide information on hydrogen bonding in peptides and proteins [18,25]. The region 1800–1500 cm^−1^ corresponds to the stretching band of amide I (C=O stretching) and the bending peak of amide II (C–N stretching and N–H bending). Considerable differences were observed in the spectra of FF@GO in comparison to FF and GO, showing that the interactions between GO and FF were mediated by hydrogen bonding (Fig. 1C). In order to confirm this, we used Raman spectroscopy for FF, GO, and FF@GO hybrid nanosheets. GO exhibits two distinct bands at 1597 (G-band) and 1357 cm^−1^ (D-band), where the G band is a distinctive feature found in the in-plane oscillation of carbon atoms connected through sp^2^ bonding and the D band is typically associated with the existence of defects in the sp^2^ bonding [26]. Raman peaks at 1002 and 1032, 1180, 1210, 1581 and 1624 cm^−1^ are associated with aromatic vibrations in FF. The amide III bands, specifically the CN bands, are observed at wavenumbers of 1236 and 1272 cm^−1^. Additionally, the amide I band, which corresponds to the C=O group, is detected at a wavenumber of 1697 cm^−1^ [27]. The highly intense bands located around 3056 cm^−1^ could be attributed to the stretching vibrations of CH and NH bonds. The intensity of the aromatic bands at 1001 and 1031 cm^−1^ is decreased in comparison to those observed for FF due to the lack of CO stretching. The aromatic bands resulting from the in-plane bending of the CH bonds in the aromatic ring are also absent in FF@GO. The amide III bands at 1236 and 1272 cm^−1^ (CN stretch and NH in-plane bending) are not evident in FF@GO spectra (Fig. 1D). This could be ascribed to the formation of new bonds in FF@GO hybrid nanosheets. The intensity ratios I_D_/I_G_ can qualitatively estimate the number of defects in these materials. I_D_/I_G_ for GO and FF@GO is 0.85 and 0.89 respectively. The rise in I_D_/I_G_ can be attributed to the deoxygenation of GO into co-assembled hybrid nanosheets, facilitated by the non-covalent interaction with FF.

**Fig. 2:**
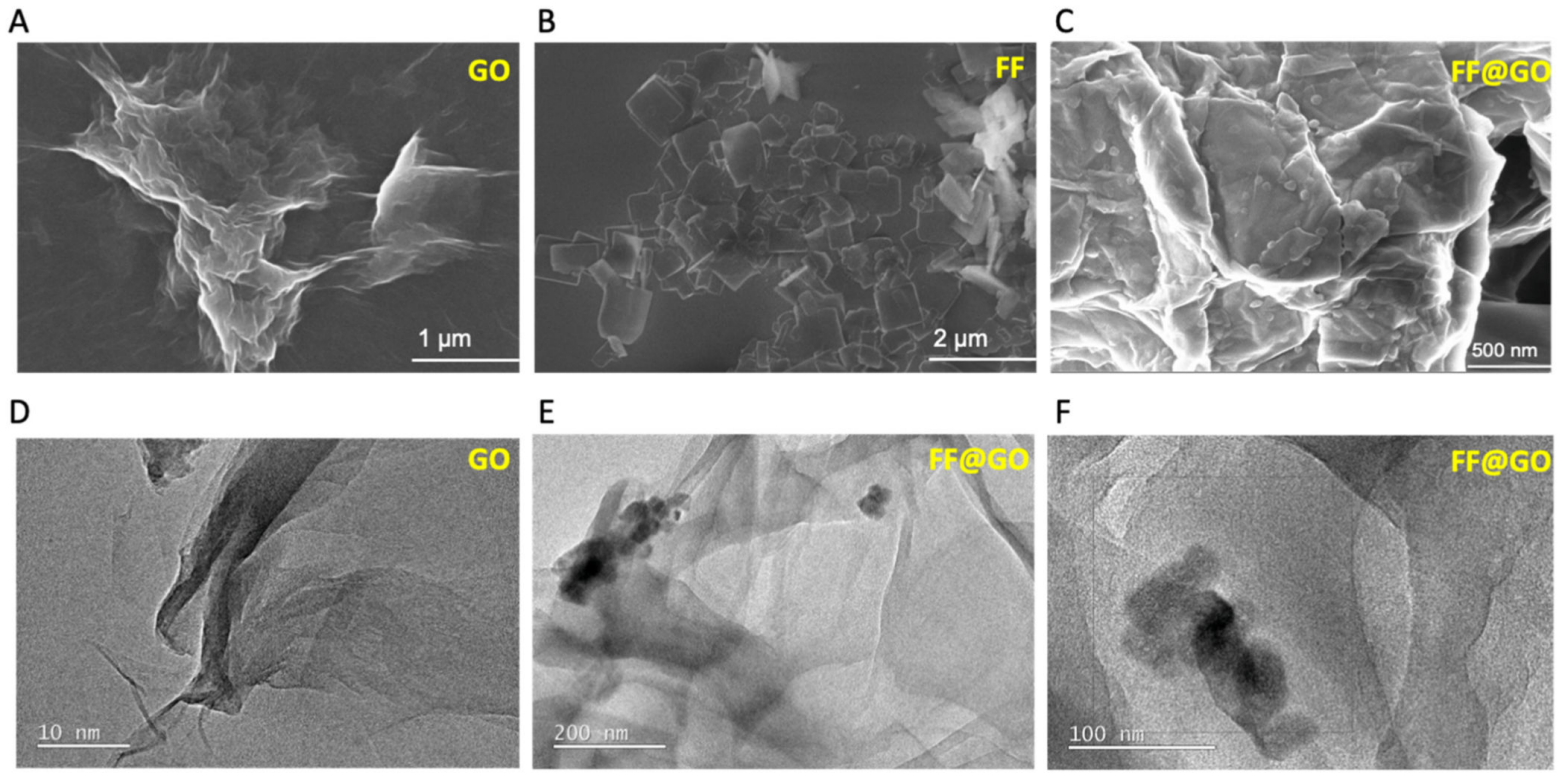
Morphological characterisation of graphene oxide (GO), FF peptide and self-assembled FF@GO hybrid nanosheets. Scanning electron microscopy (SEM) images for GO (A), FF(B) and FF@GO (C) and transmission electron microscopy (TEM) images of GO (D), and FF@GO (E, F).

Fig. 1E shows the XRD pattern of the pristine FF, GO and FF@GO. GO shows a distinct peak at 2θ ≈ 9.82 representing the (001) planes, while in the FF@GO sample, the peak is shifted to 7.7, and the spacing between layers has been increased from 0.88 to 1.16 nm, which shows the formation of the hybrid structure of FF@GO. The presence of three new peaks at 17.13°, 18.31° and 23.71° in FF@GO support the presence of FF molecules in the hybrid nanosheets. Typically, the appearance of a peak at 2θ ≈ 23.7° corresponds to lattice spacing of 0.37 nm and arises from the reduction or absence of intercalated carboxylic acid functionalities. In order to investigate the co-assembly formation between FF and GO, X-ray photoelectron spectroscopy (XPS) was carried out on GO and FF@GO samples (Fig. 1F). The XPS survey spectrum of FF@GO showed a nitrogen peak around 400 eV, whereas no N signal was detected for GO. For FF@GO, the C1s spectrum shows *C*–N and *N*–C=O bonding at 285.07 and 288.8 eV, the O1s spectrum shows *N*–O bonding at 532.4 eV, and N1s spectrum shows pyrrolic and pyridinic nitrogen, confirming the interaction between *N*-terminal amines of FF and carboxylic acids of GO in the FF@GO hybrid nanosheets (Fig. 1G–I). In order to further investigate the morphology of FF@GO hybrid nanosheets, scanning electron microscopy (SEM) and transmission electron microscopy (TEM) were used to observe the morphologies of hybrid nanosheets. The typical SEM micrograph of GO shows an interconnected network of sheets (Fig. 2A), while aggregates of FF possessed a flake-like structure (Fig. 2B). Accordingly, the SEM image of FF@GO shows the coverage of GO sheets with the FF flakes (Fig. 2C). Representative TEM images for GO (Fig. 2D) and FF@GO sheets (Fig. 2 E and F) reveal that FF did not modify the basic morphology of graphene. Taken together, the SEM and TEM images revealed smooth attachment of FF on GO nanosheets.

This bottom-up synthesis approach is simple, scalable, cost-effective, and a binder-free way to prepare co-assembled FF@GO hybrid nanosheets. An important variable that determines co-assembly and therefore the properties of the produced hybrid nanosheets is the concentration ratio of FF to GO. The results shown above are for an optimised ratio of 2.5:1 (FF:GO), which was determined experimentally by comparing Raman spectra and XRD patterns over a wide range of ratios (Supplementary Figs. 1 and 2), with additional FTIR and zeta potential measurements for the most promising formulations (Supplementary Figs. 3 and 4). When the FF concentration is high, the FF@GO structure shows no accessible GO; therefore, the analytical measurements resemble those of pure FF, and the resulting structure will be unable to store NO gas. As the amount of FF decreases, the GO volume increases and the resulting structure should be able to hold more NO gas, however, low FF concentrations will result in reduced structural stability and pH reactivity. This work indicates that the ratio of FF to GO significantly influences the development of co-assembled hybrid nanosheets.

**Fig. 3:**
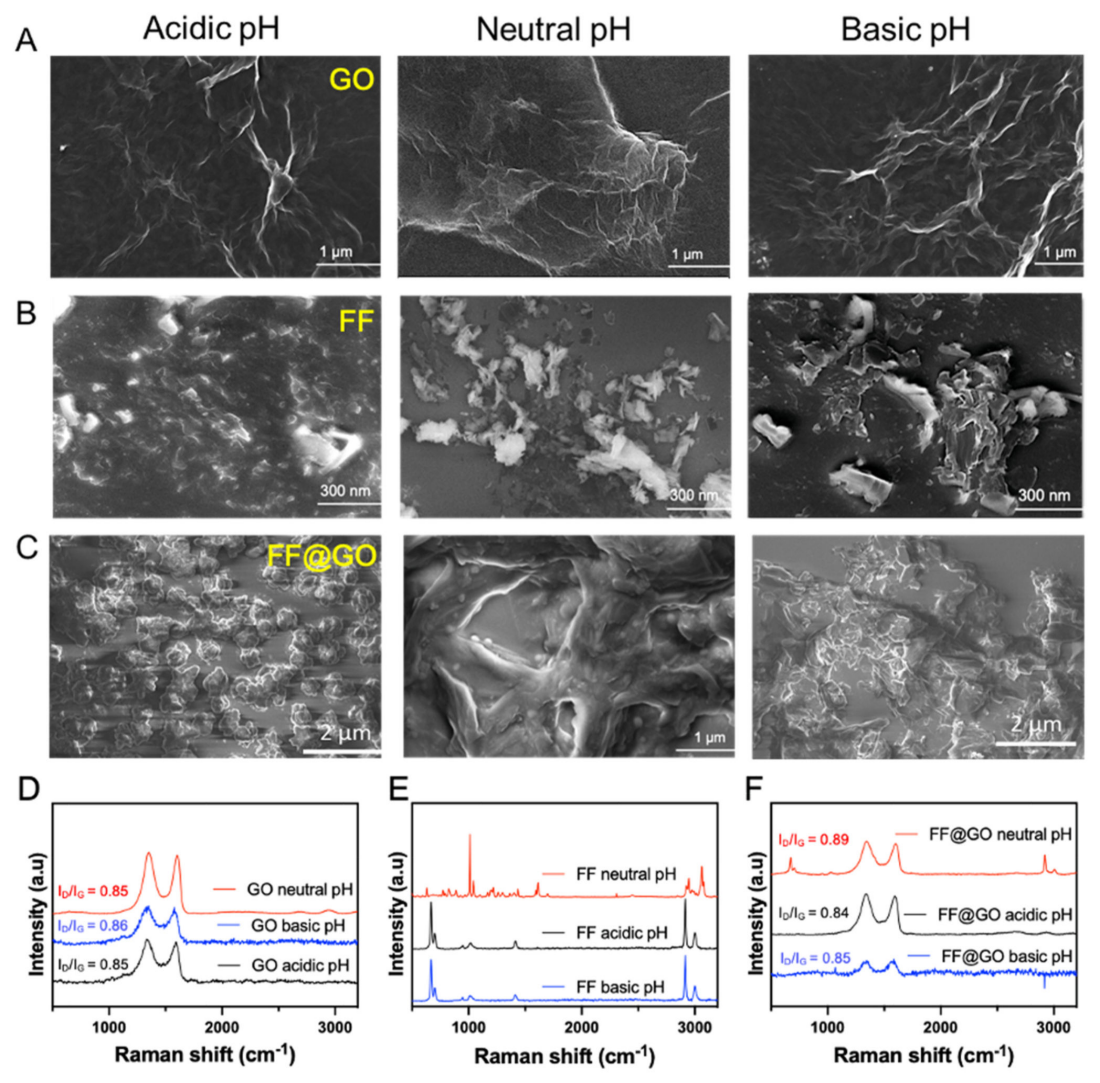
pH-responsive behaviour of the synthesised materials. SEM images of (A) GO, (B) FF, and (C) FF@GO hybrid nanosheets. pH dependent morphology shows that GO retains its morphology under neutral, acidic, and basic pH while FF@GO shows the disruption of the hybrid nanosheets. (D–F) Raman spectra of GO, FF, and FF@GO respectively under neutral, acidic (pH 4.5) and basic (pH 10) conditions.

To assess the pH-responsiveness of FF, GO, and FF@GO, the samples were protonated/deprotonated in acidic (pH-4.5) and basic (pH-10) solutions. SEM observation showed that GO and FF partially retained their morphologies under neutral, acidic, and basic pH while the co-assembled FF@GO hybrids nanosheets experienced disruption, shrinkage, and deformation under acidic conditions, due to the breaking of hydrogen bonds and subsequent protonation of the *N*-terminal amines of FF (Fig. 3A–C). These observations were confirmed by Raman spectroscopy, where GO on their own retained their spectral fingerprints, demonstrating stability under acidic and basic pH. In contrast, there was a shift in peaks in FF@GO under both acidic and basic pH (Fig. 3D–F). The I_D_/I_G_ ratio of FF@GO under acidic condition decreased from 0.89 to 0.84, which indicates repair of GO defects by breaking the π-conjugated hybrid structure in the original hexagonal carbon network. This reduction in I_D_/I_G_ ratio also indicates the disorder associated with oxygen functional groups being diminished [28]. The I_D_/I_G_ ratio for FF@GO under basic condition is 0.85 which is equal to GO. In summary, these results indicate the pH-responsiveness of FF@GO co-assembly hybrid nanosheets which was governed by de-protonation of the amide groups in FF@GO under acidic pH.

**Fig. 4:**
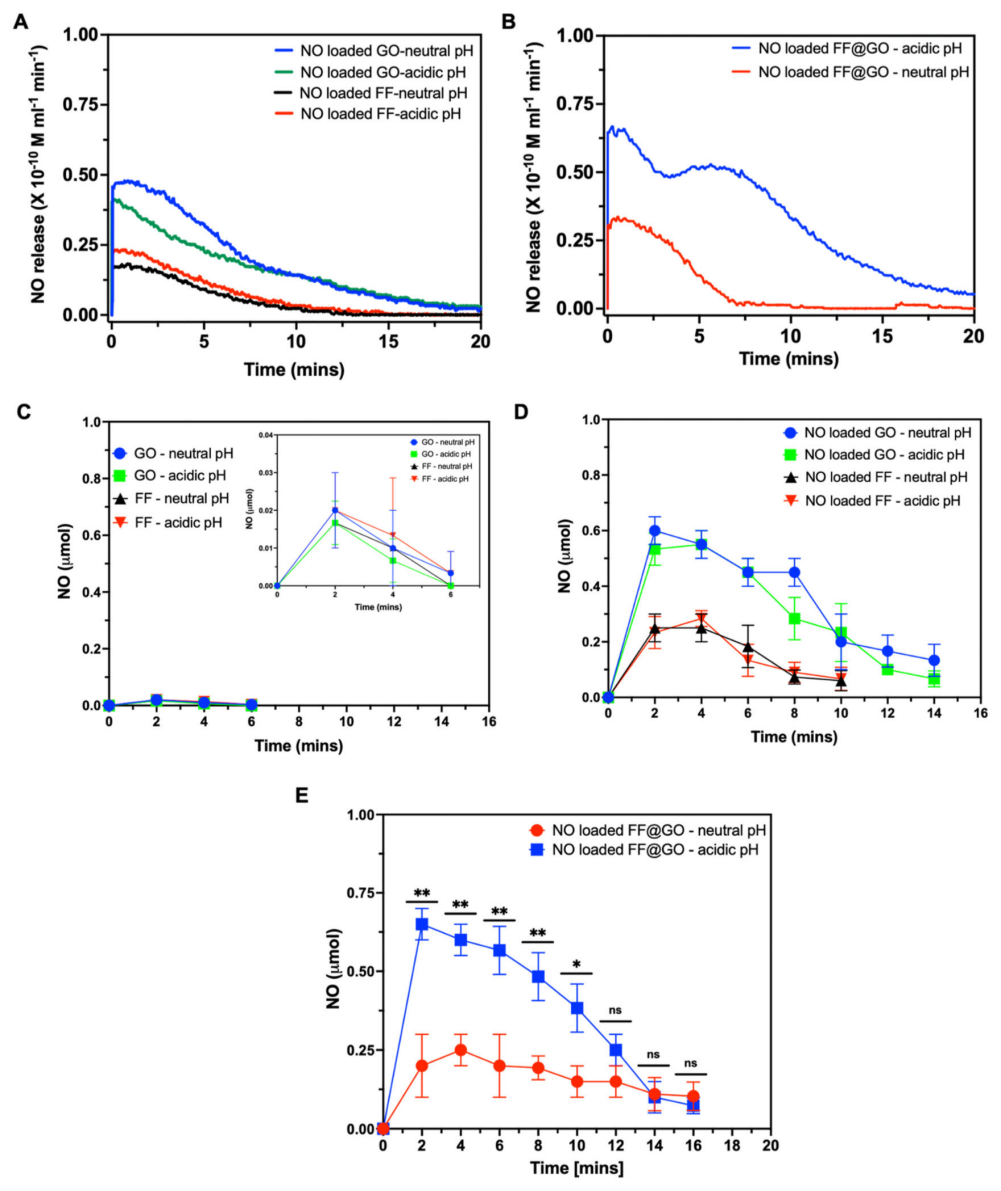
NO release from NO-loaded GO, FF, and FF@GO hybrid nanosheets at different pH levels. (A–B) Real-time NO release from different formulations measured using electrochemical sensor under neutral (pH 7.4) and acidic (pH 4.5) conditions. (C) NO release from GO and FF under neutral and acidic pH levels detected using fluorescent dye (DAF-FM). Values represent mean ± standard deviation, n = 3. (D) NO release from NO loaded GO and NO loaded FF under neutral and acidic pH detected by DAF-FM. Values represent mean ± SD, n = 3. (E) NO release from NO loaded FF@GO under neutral and acidic pH levels detected by DAF-FM. Data is presented as mean ± SD with Student’s t-test to compare neutral and acidic pH for each time-point, where ns denotes not significant, * = p < 0.05, and ** = p < 0.01.

Formulations were then loaded with NO gas and the real-time kinetics of NO release from GO, FF, and FF@GO were investigated using a NO electrochemical sensor in a pH-dependent manner at 37 °C under continuous stirring. The GO and FF peptides alone did not reveal any substantive difference between the release patterns of NO at neutral and acidic pH (Fig. 4A). The release of NO from FF@GO in acidic pH (4.5) was much more rapid and sustained compared to physiological pH (7.4). The increased release rate of FF@GO in acidic pH could be attributed to the protonation of the amine groups in FF peptide, which possess a lone pair of electrons on nitrogen. This process of protonation and deprotonation changes the apparent charge on both components that helps the disassembly of hybrid nanosheets. In addition, the formation of Schiff base during the crosslinking of the FF provides pH sensitive properties to the hybrid nanosheets, since the Schiff base bond exhibits dynamic properties and behaves differently at different pH levels. We postulate that at acidic pH (4.5) fast hydrolysis of the Schiff base and a breakdown in the electrostatic, hydrogen bonding and π-π stacking, results in a loose nanostructure allowing faster release of NO.

NO release was also quantified using a fluorescent NO-specific probe 4-amino-5- methylamino-2′,7′-difluorofluorescein (DAF-FM), which undergoes irreversible nitrosation in contact with NO resulting in the formation of a fluorescent triazole [29]. Samples of GO and FF alone (without NO loading) were tested as a control and indicate the release of trace amounts of NO which are not biologically relevant (Fig. 4C). Such trace amounts of NO may originate from atmospheric nitrogen or from reactions involving nitrogen-containing compounds and can be present in materials as an environmental contaminant or from synthesis. The samples were therefore tested without NO loading and then were degassed before loading NO gas into them. When formulations were loaded with NO gas, the NO release rates from FF and GO were unaffected by pH (Fig. 4D), while NO release from co-assembly FF@GO is triggered by acidic pH (Fig. 4E), in agreement with the electrochemical sensing data (Fig. 4 B).

**Fig. 5:**
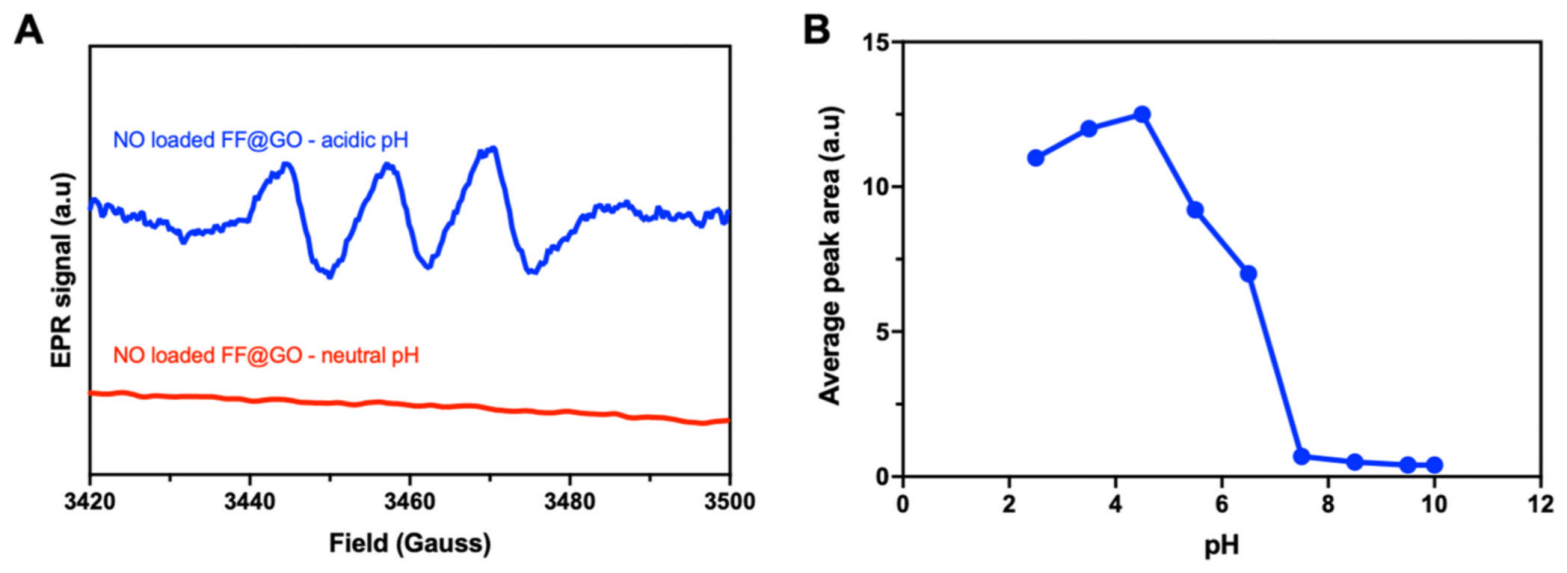
NO release from NO-loaded FF@GO (250 μg/ml) as a function of pH using X-band CW-EPR. (A) EPR of NO release from NO loaded FF@GO (under both neutral and acidic pHs) incubated with DETC_2_Fe spin trap as detected by the formation of NO–Fe(II)DETC_2_ complex (n=3) and (B) average peak area from EPR spectra obtained from NO loaded FF@GO when incubated with DETC_2_Fe spin trap at different pH (n=3).

Electron paramagnetic resonance (EPR) spectroscopy was used to directly and unambiguously confirm the release of NO from samples by detecting the paramagnetic properties of NO, which are influenced by hyperfine splitting arising from the nitrogen nucleus of NO [30]. In this study we used a (DETC)_2_Fe complex to trap NO released from samples producing the EPR active spin adduct NO-(DETC)_2_Fe. The characteristic three-line isotropic EPR spectrum of NO-(DETC)_2_Fe clearly reveals that NO gas is released from FF@GO only under acidic conditions, with an isotropic g-value of 2.04 and a clear ^14^N hyperfine coupling of 36 MHz or 1.3 mT (Fig. 5A). The NO release from FF@GO is highly pH-dependent (Fig. 5B). The pH-dependent release thus enabled good preservation of NO under neutral and high pH environment but high NO release efficiency under acidic environment, suggesting potential applications in site-specific drug delivery systems. In contrast, this signal was not produced by FF, GO, FF@GO under neutral pH, or (DETC)_2_Fe (Supplementary Fig. 5).

The synthesis of organic-inorganic hybrid nanosheets from peptide modified GO represents an important step toward the development of a pH-responsive NO-delivery system. Such stimuli-responsive systems can also be adopted to polymeric fibres. Typically, modern encapsulation methods, such as electrospinning and pressure spinning could allow for precise control over the size, shape, and morphology of encapsulated formulations. By adjusting parameters such as pressure, flow rate, and spinning speed, the properties of the encapsulated formulations can be precisely controlled to meet specific application requirements [31,32].

In the current study, the combined features of short-term NO release and pH responsiveness using a nanomaterials approach demonstrates for the first time the potential of delivering NO gas in a controlled manner. Releasing NO as a gas offers several advantages compared to releasing it from a compound, for example, NO gas is highly diffusible and can penetrate biological tissues rapidly. As a gas, NO can traverse cellular membranes and reach target sites more efficiently than large molecules or compounds which require the presence of a trigger/reducing agent to convert such compounds into NO, e.g. relying on the presence of reduced glutathione (GSH) to accelerate the decomposition of SNAP into NO via direct transnitrosation [33]. The reliance on such agent-dependent NO production may ultimately prove limiting. Furthermore, the covalent attachment of NO donors to materials requires additional synthesis steps. In contrast, materials with high surface areas and tuneable pore structures allow the direct adsorption of NO gas onto the surface and within the pores. This adsorption process can occur through physical interactions, such as van der Waals forces or electrostatic interactions, without requiring chemical modification of the material. The NO-release characteristics are controlled by a peptide layer which is reversible/switchable in biologically relevant conditions. Since NO concentration and release time dictate its biological action, such a NO-releasing delivery system may prove useful to prevent NO-release while the nanoparticles are circulating in the blood stream (neutral pH), but result in targeted release to tissues or cells under acidic conditions. For example, following a myocardial infarction, the lack of oxygen in the ischaemic cells leads to a switch from aerobic to anaerobic metabolism, resulting in the production of lactic acid and the acidification of the intracellular space [10,34]. Nanoparticles taken up by these cells would therefore preferentially release NO in this acidic environment to have their therapeutic effect and reduce ischaemia/r-eperfusion injury. Moreover, it is anticipated that a wide-range of NO-releasing kinetics could be realised by controlling the type and amount of peptide used during preparation in order to suit different therapeutic requirements. Further exploration of the applications of these novel delivery systems is currently underway.

## 3 Conclusion

We demonstrate the efficient, facile, and cost-effective synthesis of peptide-GO nanohybrids for pH-senstive release of NO gas. The co-assembly of dipeptide and graphene is achieved through a set of synergistic non-covalent interactions including π–π, electrostatic and hydrogen bonding between the two components to form hybrid nanosheets. This strategy enables the on-demand release of NO under acidic conditions, and we demonstrate that the storage of NO gas and release of NO is controllable by the pH of the local environment. The findings from this study suggest that the co-assembly of graphene with peptides could be a promising approach for use in stimuli-responsive systems for the delivery of bioactive compounds.

## 4 Materials and methods

### 4.1 Synthesis of GO and FF@GO co-assembled hybrid nanosheets

Exfoliated graphene oxide (GO) flakes were synthesised from exfoliated graphite using the modified Hummer’s method as previously reported by us [21–23]. NaNO_3_ (1.5 g) and H_2_SO_4_ (150 mL, 98 %) were added to a 800 mL round-bottom flask with graphite flakes (2 g). The flask was immersed in an oil bath after mixing the reaction mixture under magnetic stirring. Subsequently, the mixture was heated at a temperature of 35 °C, followed by the addition of 9 g of KMnO_4_ into the flask. The mixture underwent continuous stirring for 24 h, after which a further 280 mL (5 %) of H_2_SO_4_ was added and the temperature was raised to 85–95 °C. The mixture was stirred for a further 2 h before removing the oil bath. The flask was allowed to cool to 60 °C. Finally, 15 mL (30 wt%) of H_2_O_2_ was added and the mixture was stirred for another 2 h. The final product was subjected to 7–8 rounds of washing with HCl (3 %) and subsequently washed 4–5 times with distilled water to remove any impurities. As obtained GO was dispersed in water under stirring. The resulting solution was used for further synthesis and characterisation. To synthesise co-assembled FF@GO hybrid nanosheets, a fresh FF peptide stock solution was prepared by dissolving the FF powder (Bachem, Switzerland) in dimethyl sulfoxide (DMSO) (Thermo Fisher, UK). Co-assembled FF@GO hybrid nanosheets were obtained by mixing FF and GO from equal, increasing and decreasing amounts of GO and FF. The ratio at which we obtained optimally co-assembled FF@GO hybrid nanosheets was 2.5:1 mg of FF and GO. Briefly, a 1 ml of GO solution dissolved in water (1 mg/ml) was mixed with a 1 ml solution of FF (2.5 mg/ml) dissolved in DMSO in a round bottom-flask for 1 min at room temperature. The resulting solution was used for further characterisation.

### 4.2 Characterisation of FF@GO co-assembled hybrid nanosheets

As prepared GO and co-assembled FF@GO hybrid nanosheets were used for further characterisation. Samples were mounted to glow-discharged *C*-Flat 1.2/1.3 4C grids (Protochips) for transmission electron microscopy (TEM). The micrographs were obtained at ambient temperature using a 300 kV Titan Krios electron microscope that was fitted with a Falcon 3 detector (Thermo Scientific) and a Cs corrector (CEOS). To prepare the TEM samples, 2 μl of as-prepared GO and FF@GO samples were placed onto a holey carbon copper grid. X-ray diffraction (XRD) investigation was performed utilising Cu Kα radiation. X-ray measurements were conducted using a voltage of 40 kV and a current of 40 mA. The spectra were obtained with a step size of 0.02° (2θ) and a step time of 1 s. Fourier-transform infrared (FTIR) spectroscopic analysis was performed using a Tensor-27 FTIR spectrometer (Bruker Optics, Champs-sur-Marne, France) within the wavenumber range of 4000–100 cm^−1^. FTIR samples were prepared by mixing the sample with KBr. Raman spectroscopy was conducted using laser excitation at a wavelength of 532 nm (Renishaw, Stroud, UK). VG Multilab 2000 (Al-Kα, γ = 1.486 keV) machine was used to measure X-ray photoelectron spectroscopy (XPS) of samples in the high vacuum environment. Samples were spread on the Cu foil and were measured on the different spots to identify the isotropy of the sample. Final XPS spectra were analyzed using Casa-XPS software. The surface charge of GO, FF, and FF@GO solutions (0.1 mg/mL) was assessed using a dynamic light scattering analyser (Zetasizer, 2000HAS, Malvern, Worcestershire, UK).

### 4.3 Nitric oxide gas loading

Due to the rapid reaction of NO with oxygen, samples were first sparged using N_2_ gas to remove all dissolved oxygen. Samples were loaded and stored in 7 mL glass vials with PTFE liner (27151 & 27157, Sigma-Aldrich, USA). Samples were purged with a N_2_ headspace (BOC, UK) for 5 min and subsequently loaded with NO (400 ppm NO in Nitrogen, BOC, UK) for 5, 10 and 60 min. A blunt needle placed just above the liquid surface was used to control gas flow and ensure thorough sample mixing.

### 4.4 Measurement of nitric oxide gas release

Real-time NO release was evaluated by a free radical analyser (TBR4100, World Precision Instruments) equipped with an NO-sensitive electrode (ISO–NOP, World Precision Instruments). Prior to the use, the NO electrode was polarised and calibrated in accordance with the instructions provided in the manual. Specifically, the probe was submerged in a glass vial containing 10 mL of a solution consisting of 0.1 M H_2_SO_4_ and 0.1 M potassium iodide. This was done to establish a consistent current measurement. Successive aliquots of 25 μM NaNO_2_ solution were added to the mixture to create a range of NO concentrations for the calibration curve. The NO concentrations were calculated according to the amount of NaNO_2_ input as the conversion of NaNO_2_ to NO was stoichiometric equal. To detect the concentration of NO release from GO, FF peptide and FF@GO, the NO probe was immersed in a glass vial filled with 4 mL of the sample (0.25 mg/mL) in buffers having physiologically neutral (pH 7.4), acidic (pH 4.5) and basic (pH 10) pH. The calibration curve was used to determine NO release from real-time current response changes. Aluminium foil was used to cover glass vials, which were kept on a hot plate at 37 °C with continual stirring during NO measurement. NO release was also quantified by utilising DAF-FM (Abcam, UK). Briefly, 60 μL particle suspension (0.5–1.0 mg/mL) was mixed with 10 μL DAF-FM (1 μM, in PBS), and 220 μL buffer in a black clear bottom 96 well-plate. The mixture was incubated at 37 °C for 30 min and the fluorescence was measured every 2-min utilising a SpectraMax M5 microplate reader (Molecular Devices, λex/em = 495/515 nm). All statistical comparisons were performed using GraphPad Prism 9 (GraphPad Software). Comparisons between NO loaded FF@GO hybrid nanosheets under acidic pH at each time points were made using ordinary *t*-test for comparisons with the NO loaded FF@GO under neutral pH.

Electron paramagnetic resonance (EPR) was used to detect NO release from samples. DETC_2_Fe was employed for trapping of NO as reported in previous studies. NaDETC (250 mM, 5 ml) and iron (II) sulfate (FeSO_4_.7H_2_O, 50 mM, 5 ml) were separately dissolved in degassed Milli-Q water. These were rapidly mixed in 10 ml of dichloromethane (CH_2_Cl_2_) to obtain a pale yellow-brown opalescent colloid DETC_2_Fe solution, which was used immediately. Samples (at the concentrations of 250 μg/ml) were incubated with the spin trap solution for 30 min. Then an aliquot (20 μL) of the resultant solution was transferred to a quartz EPR tube and analyzed by X-band EPR spectroscopy. The room temperature EPR spectrum exhibits three lines that confirm the formation of NO-DETC_2_Fe complex. EPR spectra were collected with a conventional continuous wave (CW) homodyne microwave bridge and TE_011_ resonator. The spectrometer was a Bruker BioSpin EMXmicro with a Premium X-band (9.1–9.9 GHz) source.

The microwave frequency is 9.877 GHz, with a power of 10 mW and a field modulation of 0.3 mT. The resonator was a super high-quality Bruker BioSpin SHQE-W1 in a 0.6 T electromagnet. All measurements were accomplished at room temperature with 1.2 mm ID clear fused quartz capillaries and nitrogen gas purge. Signal amplitudes are normalised by instrumental parameters and samples filled the vertical height of the resonator. At 50 mM Fe(II)SO_4_, and 250 mM DETC the nitrosyl iron signal is located on the slope of a large ferromagnetic signal of iron oxides.

## Supplementary Material

Appendix A. Supplementary data

## Data Availability

All the data has been presented in the main manuscript and supplementary information.
