# Supplementary material for: pH-sensitive release of nitric oxide gas using peptide-graphene co-assembled hybrid nanosheets
Source: Nitric Oxide. Author manuscript; Available in PMC 2025 Aug 30. (PMC7618039; doi:10.1016/j.niox.2024.04.008)
Supplement: Appendix A. Supplementary data [file EMS207961-supplement-Appendix_A__Supplementary_data.docx]

**Supporting information**

# pH-sensitive release of nitric oxide gas using peptide-graphene co-assembled hybrid nanosheets

Tanveer A. Tabish^1^, Jiamin Xu^2^, Christopher K. Campbell^3^, Manzar Abbas^4^, William K. Myers^5^, Pravin Didwal^6^, Dario Carugo^3^, Fang Xie^2^, Mark J. Crabtree^1,7^, Eleanor Stride^8^, Craig A. Lygate^1^

^1^Division of Cardiovascular Medicine, Radcliffe Department of Medicine, British Heart Foundation (BHF) Centre of Research Excellence, University of Oxford, Oxford, OX3 7BN, United Kingdom

^2^Department of Materials and London Centre for Nanotechnology, Imperial College London, London, SW7 2AZ, United Kingdom

^3^Nuffield Department of Orthopaedics, Rheumatology and Musculoskeletal Sciences (NDORMS), The Botnar Research Centre, University of Oxford, Oxford, OX3 7LD, United Kingdom

^4^Department of Chemistry, Khalifa University of Science and Technology, P.O. Box, 127788, Abu Dhabi, United Arab Emirates

^5^Centre for Advanced Electron Spin Resonance (CAESR), Inorganic Chemistry Laboratory, Department of Chemistry, University of Oxford, Oxford, OX1 3QR, United Kingdom

^6^Department of Materials, University of Oxford, Parks Road, Oxford, OX1 3PH, United Kingdom

^7^Department of Biochemical Sciences, School of Biosciences and Medicine, University of Surrey, Guildford, GU2 7XH, United Kingdom

^8^Institute of Biomedical Engineering (IBME), Department of Engineering Science, University of Oxford, Oxford OX3 7LD, United Kingdom

**Corresponding author:** Tanveer A. Tabish, email: [tanveer.tabish@cardiov.ox.ac.uk](mailto:tanveer.tabish@cardiov.ox.ac.uk)

**Chemicals and reagents**

Sodium nitrate (NaNO_3_), sulfuric acid (H_2_SO_4_ - 95.0–98.0%), potassium permanganate (KMnO_4_), hydrogen peroxide (H_2_O_2_ - 30 wt%), and hydrochloric acid (HCl - 36 wt%), were purchased from Thermo Scientific, Fisher Scientific, Acros, Nacalai Tesque and Alfa Aesar, respectively. Graphite flakes, phosphate buffered saline (PBS), sodium nitrite (NaNO_2_ - 97%), potassium iodide, and transmission electron microscopy (TEM) copper grid, diethyldithiocarbamic acid sodium salt (DETC), diethyldithiocarbamic acid sodium salt (DETC), iron(II) sulfate heptahydrate (FeSO4•7H2O, [≥99%](https://www.sigmaaldrich.com/GB/en/product/sigald/f7002)) and dicholormethane (CH_2_Cl_2_) were purchased from Sigma Aldrich. 4-amino-5- methylamino-2′,7′-difluorofluorescein (DAF-FM) was purchased from Abcam, UK. All materials were used as received without any further purification unless stated otherwise.

**FF@GO co-assembled hybrid nanosheets**

FF@GO hybrid nanosheets were analysed at different ratios of FF and GO using various spectroscopic methods to gain a deeper understanding of the conjugation mechanisms.

**The effect of FF to GO ratios on Raman spectra**

It is critical to optimise the FF and GO concentration combinations to make FF@GO co-assembled hybrid nanosheets. First, we performed Raman spectroscopy. With increasing concentrations of FF, the FF@GO structure shows no accessible GO, while on the other hand, with decreasing concentrations of FF, the FF@GO structure does not display the features of a hybrid structure arising from GO and FF peaks (Figure S1). Also, FF@GO structure with less FF will result in less structural stability and less pH reactivity. To investigate the hybrid structure, we further performed XRD.


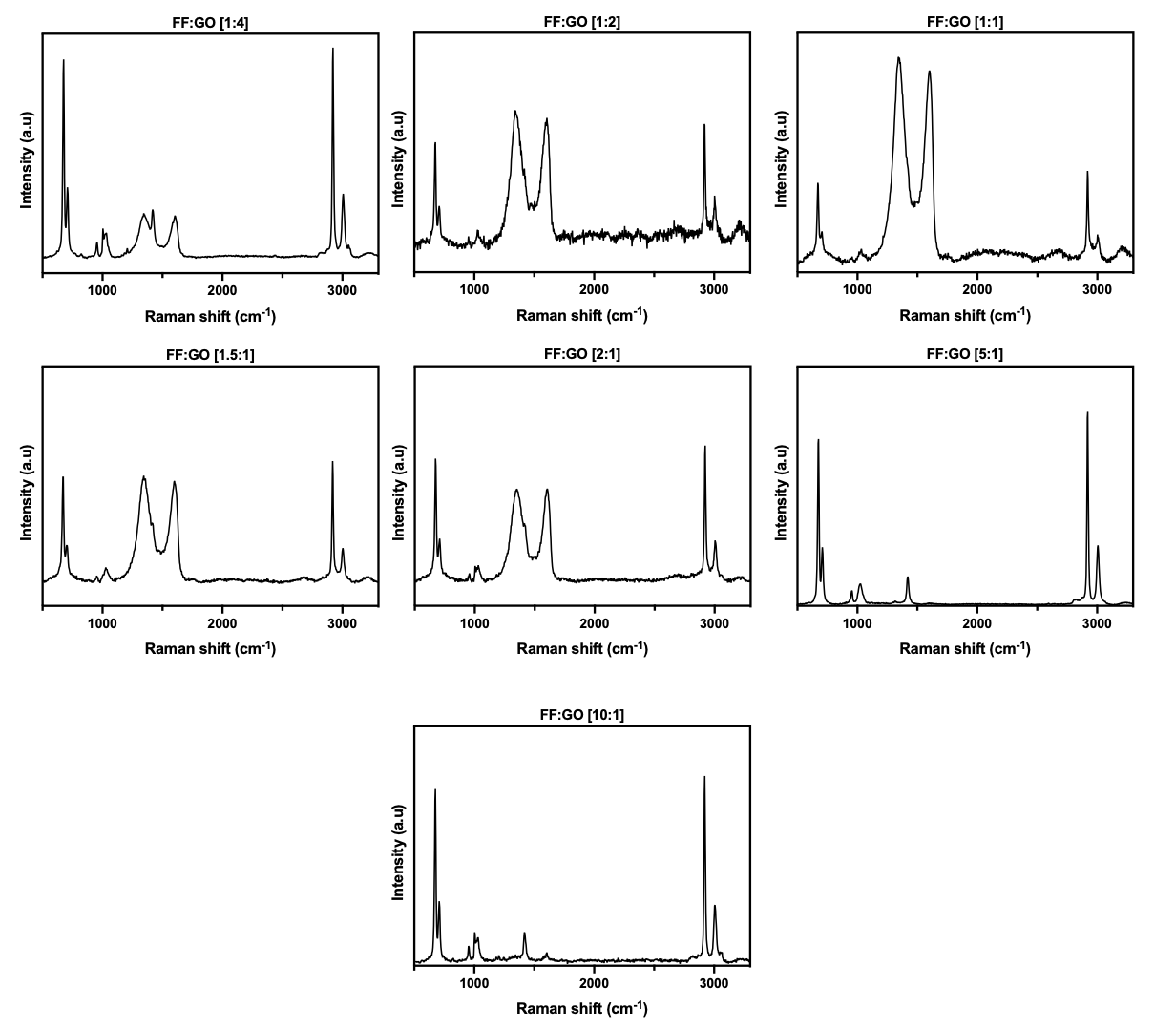


**Figure S1**: Raman spectra of FF@GO at different ratios of FF and GO.

**XRD analysis**

For FF@GO nanosheets with low FF concentrations, the XRD peaks do not show the representative peaks of a hybrid structure, such as the high-intensity peaks at 2θ ≈ 12 do not show the increase in interlayer spacing between GO sheets. Also, the appearance of multiple peaks at 2θ ≈ 27 and 32 are similar to FF suggesting that the low concentrations of FF do not bind with GO. The increasing concentrations of FF in FF@GO reveal the presence of multiple peaks arising from pure FF suggesting that FF has not been successfully conjugated with GO. The presence of such peaks suggests that FF is not reacting with GO. An optimised ratio of 2.5:1 (FF:GO), which was determined experimentally by comparing Raman spectra and XRD patterns shows the formation of FF@GO co-assembled hybrid nanosheets. We therefore used the most promising concentrations for FTIR analysis.


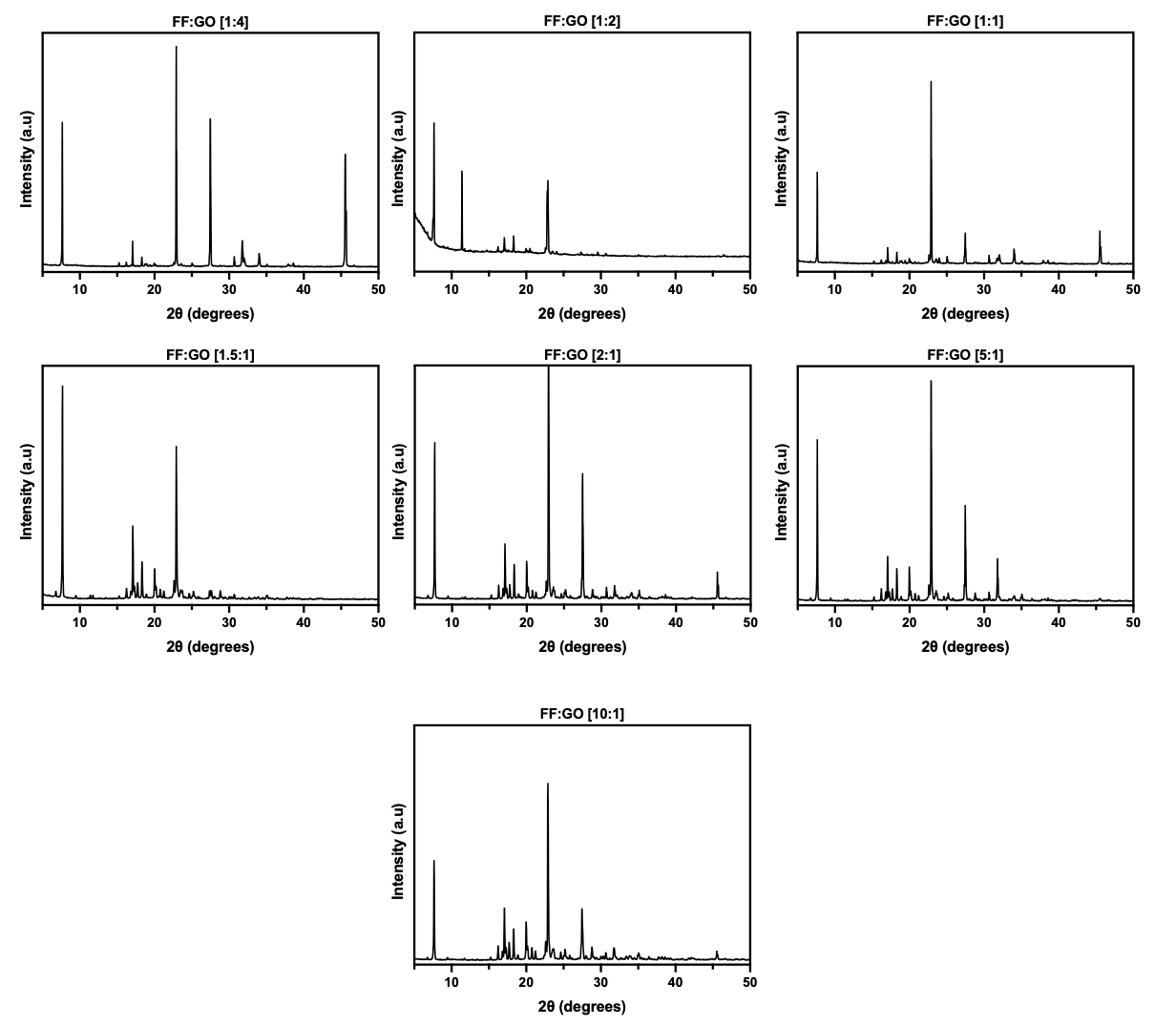


**Figure S2**: XRD patterns of FF@GO at different ratios of FF and GO.

**FTIR analysis**

The FTIR region 3500–3200 cm^−1^ is important to evaluate the hydrogen bonding arising from N–H stretching vibrations. The FF and GO at the ratios of 1:2 and 2:1 did not reveal any hydrogen bonding in this region. These peaks show that the conjugation of FF with GO was not successful at these concentrations.


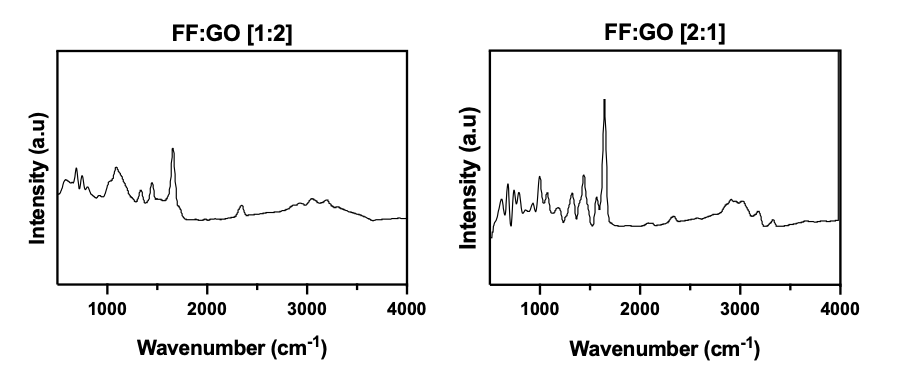


**Figure S3**: FTIR spectra of FF@GO at different ratios of FF and GO.

**Zeta potential analysis**

Electrostatic interactions between different ratios of FF and GO show that the ratios of 1:2 and 1:1 (FF:GO) had a minimal effect on the surface charge of FF@GO in comparison to that of pure GO at -18.73.


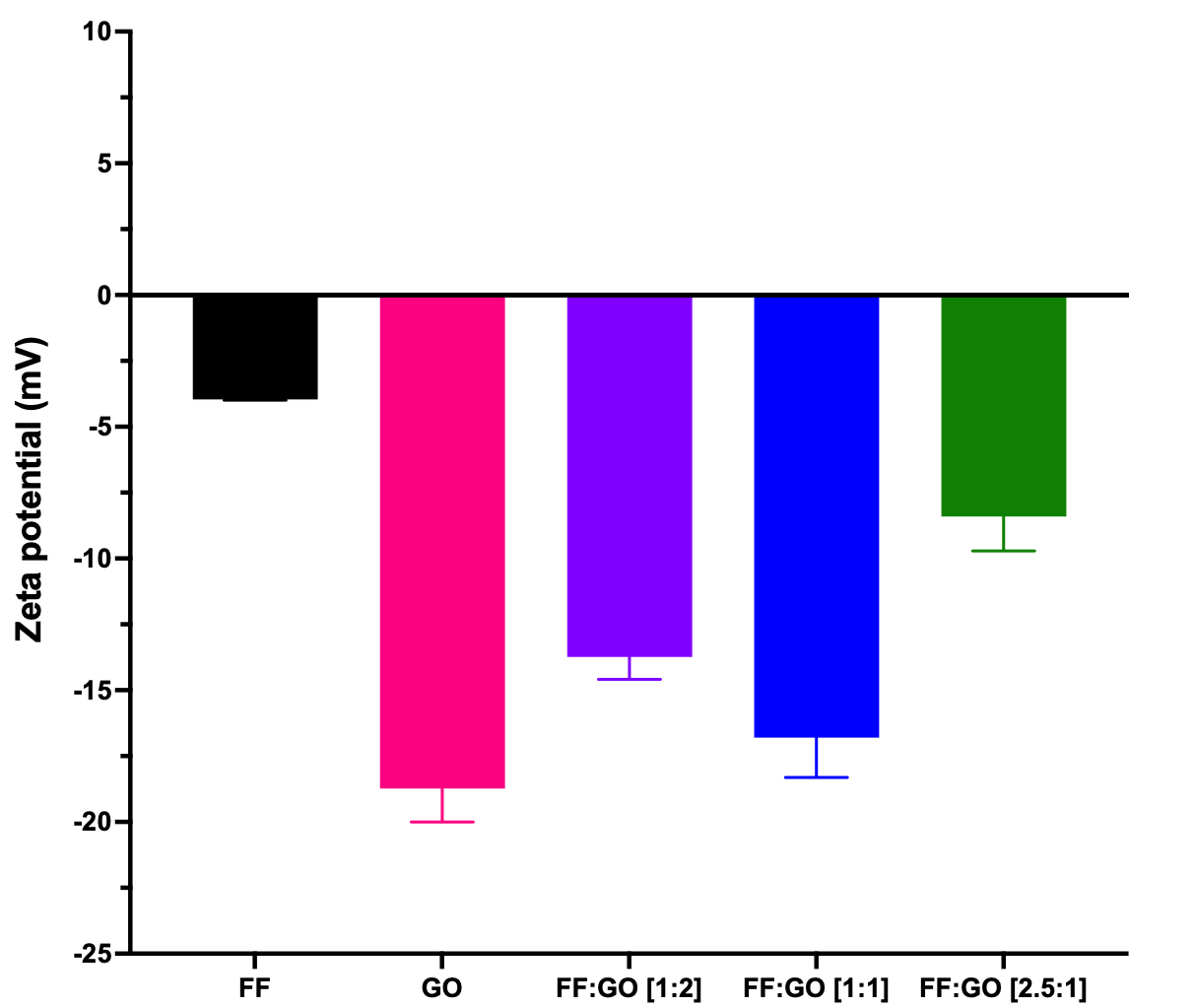


**Figure S4**: Zeta potential of FF, GO and FF@GO at different ratios of FF and GO, n=3.

**NO detection by EPR**

The characteristic three-line isotropic EPR spectrum of NO-(DETC)_2_Fe clearly reveals that NO gas is released from FF@GO only under acidic conditions. In contrast, no comparable signal was produced by unloaded FF, GO and (DETC)_2_Fe, or by NO-loaded FF@GO under neutral pH.


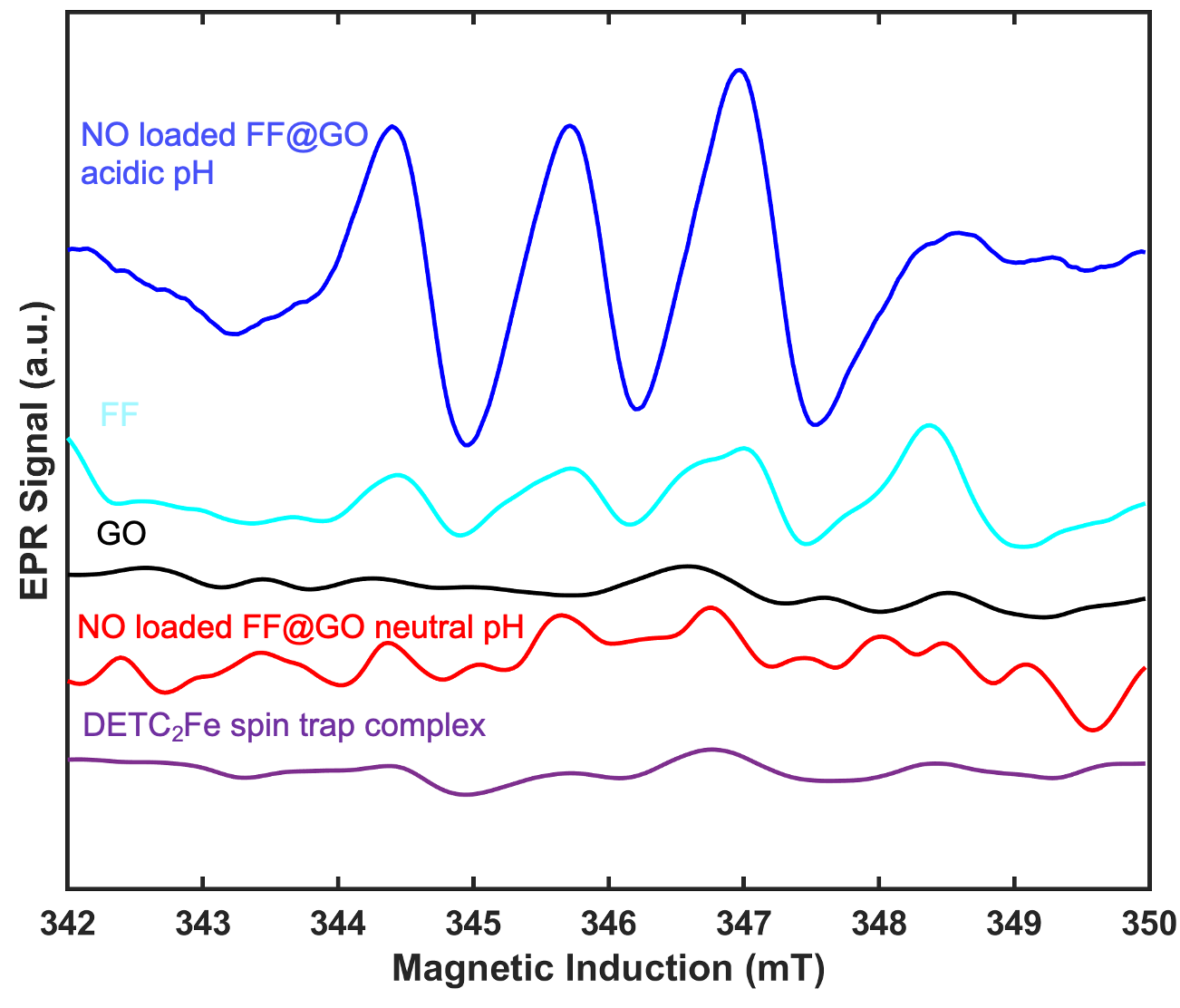


**Figure S5**: X-band CW-EPR of NO release from FF, GO, and NO-loaded FF@GO (under both neutral and acidic pH) at the concentrations of 250 μg/ml and DETC­_2_Fe spin trap as detected by the formation of NO-Fe(II)DETC­_2_ complex (n=3).
